# AuNPs for identification of molecular signatures of resistance

**DOI:** 10.3389/fmicb.2014.00455

**Published:** 2014-08-28

**Authors:** Bruno Veigas, Alexandra R. Fernandes, Pedro V. Baptista

**Affiliations:** ^1^Nanotheranostics, Centro de Investigação em Genética Molecular Humana, Departamento de Ciências da Vida, Faculdade de Ciências e Tecnologia, Universidade Nova de LisboaCaparica Portugal; ^2^Centro de Investigação em Materiais, Departamento de Ciências de Materiais, Faculdade de Ciências e Tecnologia, Universidade Nova de LisboaCaparica, Portugal; ^3^Centro Química Estrutural, Departamento de Ciências da Vida, Faculdade de Ciências e Tecnologia, Universidade Nova de LisboaCaparica, Portugal

**Keywords:** nanotechnology, nanodiagnostics, gold nanoparticles, AuNPs, multidrug resistance, Tuberculosis, molecular diagnostics technologies

## Abstract

The increasing levels of drug resistance are one of biggest threats to overcome microbial infection. The ability to rapidly and accurately detect a given pathogen and its drug resistance profile is essential for the appropriate treatment of patients and for preventing further spread of drug-resistant strains. The predictive and informative value of these molecular markers needs to be translated into robust surveillance tools that correlate to the target and extent of resistance, monitor multiresistance and provide real time assessment at point-of-need. Rapid molecular assays for the detection of drug-resistance signatures in clinical specimens are based on the detection of specific nucleotide sequences and/or mutations within pre-selected biomarkers in the genome, indicative of the presence of the pathogen and/or associated with drug resistance. DNA and/or RNA based assays offer advantages over phenotypic assays, such as specificity and time from collection to result. Nanotechnology has provided new and robust tools for the detection of pathogens and more crucially to the fast and sensitive characterisation of molecular signatures of drug resistance. Amongst the plethora of nanotechnology based approaches, gold nanoparticles have prompt for the development of new strategies and platforms capable to provide valuable data at point-of-need with increased versatility but reduced costs. Gold nanoparticles, due to their unique spectral, optical and electrochemical properties, are one of the most widely used nanotechnology systems for molecular diagnostics. This review will focus on the use of gold nanoparticles for screening molecular signatures of drug resistance that have been reported thus far, and provide a critical evaluation of current and future developments of these technologies assisting pathogen identification and characterisation.

## THE CASE FOR MOLECULAR CHARACTERISATION OF PATHOGENS

Rapid and specific detection and characterisation of agents involved in infection is of paramount importance to deliver successful treatment. Traditional methodologies for identification of pathogens, though of extreme relevance, may be laborious and time-consuming, which may lead to delayed definitive diagnoses and treatment to the patient (Mothershed and Whitney, 2005). Several innovative approaches have already made their way to the clinics and provide for increased sensitivity and specificity pathogen detection and characterisation and doing so in a fast multiplexed (Hauck et al., 2010). Additionally, the literature is full of new concepts for nucleic acid-based tests (NATs) for bacteria detection that may still make their way to the clinical setting.

Nucleic acid-based tests can be used to detect the presence of organisms directly in clinical specimens without the need of culture. In addition, hospital infection control and epidemiology programs are benefiting from the use of NATs for detecting antibiotic resistance genes and for subtyping bacteria. The first NAT cleared for use by the Food and Drug Administration (FDA) was the Gen-Probe PACE test (1988) that used nucleic acid hybridisation to detect *Chlamydia sp.* and *Gonococci*. Introduction of PCR allowed the development of a plethora of diagnostic approaches for clinically relevant bacterial pathogens (Mothershed and Whitney, 2005). One such example, already in the market, is the line probe assay (LiPA) from Innogenetics (Gent, Belgium). Innogenetics produces several line probe NATs for bacterial detection including ones for *Mycobacterium tuberculosis* complex and *Mycobacterium spp*., *rpoB* gene mutations conferring rifampicin resistance, and *Treponema pallidum* antibodies. The INNO-LiPA Rif. TB test detects the *M. tuberculosis* complex (MTBC), specifically five genotypes corresponding to sensitivity to rifampicin and four resistant genotypes. While there is great potential of molecular assays to increase the speed and accuracy of bacterial identification in the clinical laboratory, limitations of NATs must be considered. For example, sample preparation and DNA purification from complex media constitutes a serious drawback for these assays since quantity and quality of template/target is one of the main aspects that affect performance. Also, costs associated to specialized training for personal and sophisticated equipment pose a serious obstacle for the widespread implementation of NATs as front line diagnostics.

## NANOTECHNOLOGY FOR MOLECULAR DIAGNOSTICS (NANODIAGNOSTICS)

In the last decade, the use of nanomaterials for biosensing has been having a great impact and presents a great opportunity to develop fast, accurate and cost effective approaches for detection of pathogenic infectious agents. Nanodiagnostics have focused on the design of systems where researchers manipulate the properties of nanostructures for diagnostics purposes. Compared to standard methodologies, nanotechnology based approaches have several important practical advantages, including: enhanced surface reactivity, quantum confinement effects, enhanced electrical conductivity and enhanced magnetic properties, which enable nucleic acid detection to be extremely sensitive (Kaittanis et al., 2010; Chi et al., 2012; Shinde et al., 2012; Hartman et al., 2013). It should be mentioned that several systems incorporating small peptide and/or protein recognition moieties have also been reported, but fall outside the scope of the present review (for additional insights please refer to Larguinho and Baptista, 2012).

Despite the wide range of nanoscale systems for biomolecular assays (Azzazy et al., 2006; Jain, 2007; Das et al., 2010), the most promising approaches are based on nanoparticles (NPs; Rosi and Mirkin, 2005; Jain, 2007; Baptista et al., 2008; Branton et al., 2008; Tallury et al., 2009; Jung et al., 2010; Chi et al., 2012; Wang et al., 2013; Lin et al., 2014). In particular, the unique properties of noble metal NPs, such as gold, have allowed for the development of new biosensing platforms, offering greater sensitivity than conventional reporter molecules (Azzazy and Mansour, 2009). Surface chemistries of AuNPs can be easily tuned and functionalised with organic thiol molecules or thiol-containing polymers, leading to the formation of relatively strong covalent bonds (Kaittanis et al., 2010). For example, gold nanoparticles (AuNPs) conjugated with specific oligonucleotides can sense complementary DNA strands in a nearly one-on-one interaction between the NP and the target DNA molecule (Baptista et al., 2005; Jain, 2005; Azzazy et al., 2006, 2007; Veigas et al., 2012a). AuNPs’ simplicity and versatility have attracted considerable attention towards the development of molecular diagnostic applications and are becoming a critical component of nanotechnology-based detection of pathogens (Liu, 2006). AuNPs support multiple detection platforms, i.e., a target analyte can be sensed through more than one detection methodology, such as spectroscopic, colorimetric, fluorimetric and electrochemical methods (Jung et al., 2010; Upadhyayula, 2012).

**FIGURE 1:**
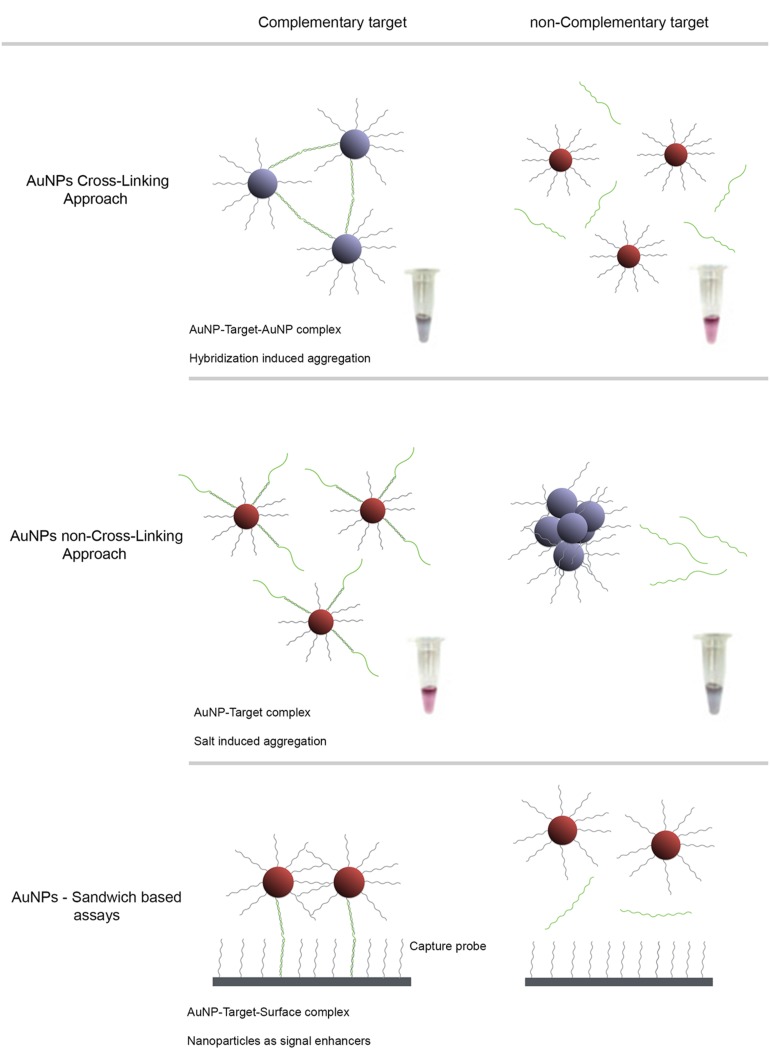
**AuNP detection schemes for characterization of antibiotic resistant pathogen strains.** Detection strategies based on the observable colorimetric alteration of Au-nanoprobes solutions. The surface plasmon band (SPR) of gold nanoparticles depends on inter-particle distance, size and aggregation. Aggregation of AuNPs results in a red-shift of the SPR with concomitant change of color of the solution, from red to blue. **(A)** Cross-Linking method relies on the hybridization of two populations of Au-nanoprobes that bind to adjacent regions of a nucleic acid target. Aggregation mediated by DNA hybridization results in a visual change of color from red to blue. **(B)** Non-cross-linking method relies on the differential aggregation profiles of Au-nanoprobes induced by increased ionic strength in the presence or absence of the specific target sequence: presence of the complementary target sequence to that of the probe prevents aggregation and the solution remains red, whereas absence of a specific target sequence leads to extensive aggregation after salt addition and the solution turns blue. **(C)** Sandwich based assay involves an immobilized capture probe, target DNA and one Au-nanoprobe reporter. Half of the target DNA hybridizes to the immobilized DNA and the other half to the reporter Au-nanoprobe. This strategy allows a simple to preform surface wash, thus increasing the signal to noise ratio and consequently the sensitivity. This method enables a higher sensitivity when coupled to an improved hybridization method that facilitates probe-target binding in a homogeneous format.

Gold nanoparticles have unique optical properties associated with a well-defined surface plasmon resonance (SPR) band in the visible region of the spectrum (Halfpenny and Wright, 2010), strongly correlated to composition, shape and inter-particle distance (Johnson et al., 2007). For example, AuNP aggregation leads to a pronounced color transition from red to blue due to plasmon coupling between NPs (Jain, 2007). Consequently, most AuNPs based methods rely on these colorimetric changes of the colloidal solution upon aggregation derived from changes to the media dielectric and/or due to recognition of a specific target. These detection strategies typically depend on the interaction between nanostructure-bound oligonucleotides and the target molecule mediated by a recognition element, which, for DNA/RNA assays, is an oligonucleotide sequence – gold nanoprobe (Au-nanoprobe). A specific complementary target may hybridize to the Au-nanoprobes and promote inter-particle cross-linking aggregation (e.g., using two Au-nanoprobes each functionalised with one half of a contiguous target recognition sequence) or stabilize nanoprobes against changes to the media dielectric (e.g., pH, ionic strength). In the latter, hybridisation of Au-nanoprobes to the target sequence will prevent the non-cross-linking aggregation induced by increasing ionic strength (**Figure 1**; Sato et al., 2003; Baptista et al., 2005). Thus, modulation of AuNP or Au-nanoprobe inter-particle distance allows control over their corresponding aggregation and dispersion levels providing visual detection for a wide range of biological entities (Hauck et al., 2010; Ngo et al., 2011).

### NANODIAGNOSTICS FOR PATHOGENS

Identification of pathogens based on specific target sequences has become the corner-stone of molecular based approaches to discriminate between organisms and characterize particular variations at the genomic level that provide unique nucleic acid signatures suitable for diagnostics. This trend has had a new surge with the development of numerous nanoparticle-based approaches designed to identify those pathogen signatures with extra sensitivity and faster than ever before (Costa et al., 2010; Kaittanis et al., 2010; Chi et al., 2012; Veigas et al., 2012a). In fact, the increase in sensitivity without loss of selectivity and specificity has promoted nanodiagnostics in the field of pathogen characterisation. Also, due to the minute dimensions of the signal transduction label (e.g., AuNPs), these systems show a high degree of miniaturization that makes them suitable for use at point-of-care or point-of-need.

The first proof-of-concept for nanodiagnostics using AuNPs was introduced by Mirkin et al. (1996) who successfully demonstrated that 13 nm AuNPs functionalized with a specific oligonucleotide sequence selectively assembled in the presence of a complementary target DNA. The development of this proof-of-concept led to the development of the first Au-nanoprobe based pathogen diagnostic system, allowing the detection of anthrax via a specific lethal factor DNA sequence (Bailey et al., 2003). This approach was further explored in a multitude of targets and samples (for further insights see Kaittanis et al., 2010; Veigas et al., 2012a and references therein).

Following a similar technological approach, Baptista et al. (2006) developed a rapid and relatively low cost method for DNA detection and generated the first application of AuNPs for the molecular diagnostics of *Mycobacterium tuberculosis* (Mtb). The method consists in differential stabilization of Au-nanoprobes in presence of DNA targets following salt induced aggregation: presence of a complementary target prevents nanoprobe aggregation and the solution remains red; whereas non-complementary/mismatched targets do not prevent gold nanoprobe aggregation, resulting in a visible change of color from red to blue. The methodology was tested in clinical samples demonstrating high efficiency with results comparable to those attained via commercial molecular tuberculosis (TB) diagnostics test, such as INNO-LiPA Rif. TB (Veigas et al., 2012a). Similar approaches have been used by Liandris et al. (2009) who developed a non-cross-linking approach for the detection of TB without the need of target amplification. Following a cross-linking approach, Soo et al. (2009) designed a set of gold nanoprobes to specifically hybridize with target DNA from Mtb strains. This methodology was evaluated by directly and simultaneously detecting *M. tuberculosis* complex (MTBC) and Mtb in 600 clinical strains.

*Staphylococcus aureus* is also one of the most important human pathogens, causing more than 500,000 infections in the US each year (Chang et al., 2013). By using aptamers that specifically recognize *S. aureus,* Chang et al. (2013) developed an ultrasensitive aptamer-conjugated-AuNPs for rapid bacterial detection. Their non-polymerase chain reaction (PCR)-based method measures the resonance light-scattering signal of aptamer-conjugated AuNPs to detect a single cell within 1.5 h. Accordingly to the authors this platform technology has the potential to develop a rapid and sensitive bacterial testing at point-of-care (Chang et al., 2013).

### GOLD NANOPARTICLES CHARACTERISATION OF ANTIBIOTIC RESISTANCE PROFILES

For the past 20 years there has been an increase in the emergence of antibiotic-resistant microorganisms with elevated pathogenesis at the global level, leading to an urgent need for new and improved approaches for bacterial quantification and identification (Pissuwan et al., 2010). **Table 1** summarizes existing AuNP-based technologies for the antibiotic susceptibility characterization of pathogens. Particularly problematic drug resistant bacteria include the methicillin-resistant strains of *S. aureus* (MRSA), responsible for many opportunistic infections, enteropathogenic *Escherichia coli*, Mtb showing multidrug resistance (MDR-TB and XRD-TB), and *Streptococcus pneumoniae*. The prevalence of drug resistant strains of Mtb (MDR and XDR-TB) and MRSA have demonstrated the need for the development of drug susceptibility systems that are capable of delivering an unequivocal response to identify with high sensitivity and in a cost-efficient manner the pathogen’s resistance profile and allowing fast and accurate therapeutic approach (Kaittanis et al., 2010).

**Table 1:** AuNPs-based systems for pathogen antibiotic susceptibility characterization.

Application	Description	Target(s)	Reference(s)
	Colorimetric detection of AuNPs spotted onto an illuminated glass waveguide Detection relies on the evaluation of SPR change upon target hybridization	Detection of *mecA* gene associated with methicillin resistance, in *S. aureus* and *S. aureus* 23S rRNA. Validation with clinical samples	Storhoff et al. (2004), Chan et al. (2014)
Antibiotic susceptibility characterization	Colorimetric detection with AuNPs. Detection relies on the evaluation of SPR change upon aggregation and the concomitant colorimetric changes that can be assessed by the naked eye	Detection of *rpo*B mutations associated Rifampicin resistance. Integration with isothermal DNA amplification strategies	Veigas et al. (2010, 2013)
	Colorimetric detection with AuNPs. Detection relies on the evaluation of SPR change upon aggregation. Sandwich hybridization assay, AuNPs act as SPR signal enhancers	Detection of *rpo*B and *inhA* mutations associated with Rifampicin and Isoniazid resistance. Integration with surface-anchored rolling circle amplification for isothermal DNA amplification. Validation with clinical samples	Xiang et al. (2013), Pedrosa et al. (2014)

**FIGURE 2:**
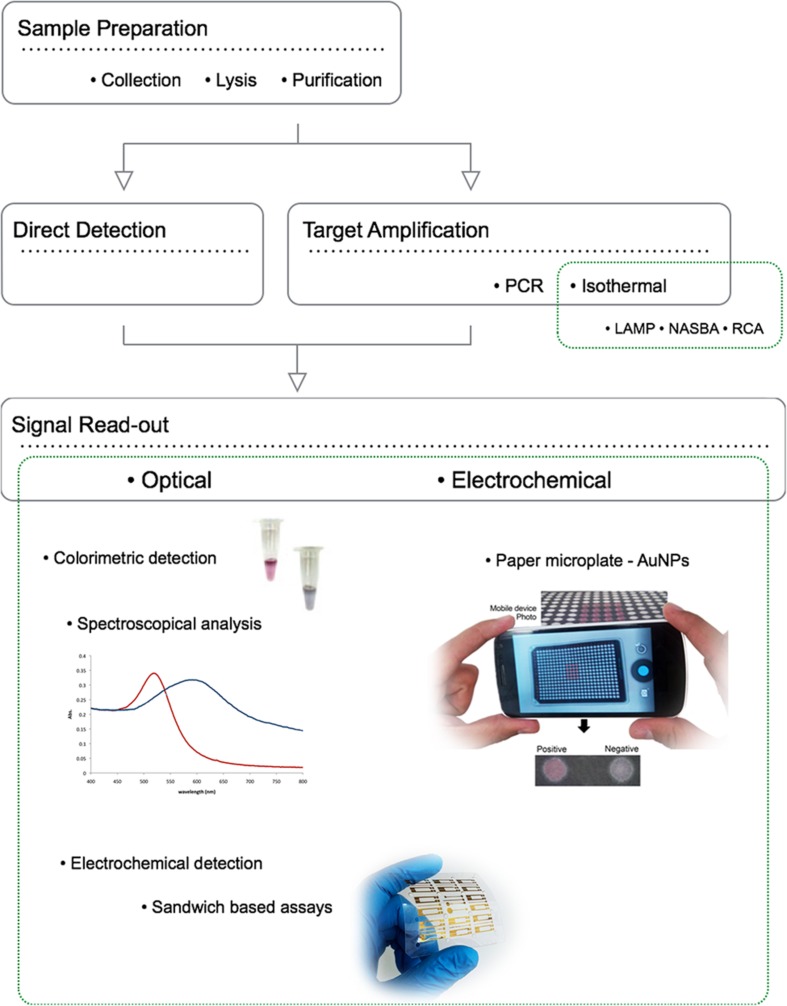
**Operational steps for pathogen detection and characterization using NPs: sample preparation, target amplification/direct detection, and signal read-out.** The ideal portable detection method should perform as accurately as traditional centralized laboratory-based testing, while also overcoming the additional challenges associated with POC testing, such as uncontrolled environmental conditions such as inconsistent or non-existent electrical power supply, and operation by untrained or minimally trained personnel. To overcome all the technical limitations of actual diagnostic systems, three distinct operational steps are required: faster simple and incorporated sample preparation, target amplification, and signal read-out. Although each step can be individually considered, a key challenge for development of POC nucleic acid detection methods is integration of all these steps into a unified process and preferably within a single device.

New diagnostic tools for drug resistant pathogen detection and characterisation ought to overcome the main constraint in terms of current molecular diagnostics – time. Several new technologies are currently being developed and validated to provide faster and at a low cost diagnosis of resistant pathogens comparing to conventional culture and drug susceptibility tests. Three distinct operational steps that are typically required for pathogen detection and characterization: sample preparation, target amplification, and signal read-out. Although each step can be considered individually, it is important to emphasize that a key challenge for development of such nucleic acid detection methods is the integration of all these steps into a unified process workflow (**Figure 2**). Rapid and cost effective diagnosis will have several benefits: earlier treatment of patients, reduction of time spent on inappropriate and ineffective treatment (thereby promoting the development of further drug resistance), and reduction of resistant strains spreading in congregate settings (Veigas et al., 2012a).

#### AuNPs for molecular detection of antibiotic resistance in *S. aureus*

Methicillin-resistant strains of *S. aureus* is responsible for 40–60% of all *S. aureus* infections in hospitals in the United States and United Kingdom (Pelgrift and Friedman, 2013). Currently, the two major strategies for laboratory detection of MRSA are bacterial culture-based phenotypic methods and NATs (Chastre et al., 2014). While a bacterial culture-based phenotypic approach offers the advantages of detecting live bacteria providing antimicrobial susceptibilities and a variety of specimens, they require 1–2 days for the confirmation of MRSA infection, due to their dependence on bacterial growth. In contrast, nucleic acid detection assays allow rapid and sensitive detection of MRSA-specific sequences directly from clinical specimens with a turnaround time ranging from 1 to 3 h (Bischof et al., 2009; Arbefeville et al., 2011; Kelley et al., 2011; Chadwick et al., 2013; Chastre et al., 2014). Combining NATs and AuNPs in a single system allows the identification the pathogen’s resistance profile with high sensitivity and in a cost-efficient manner providing fast and accurate therapeutic approaches (see **Table 2**).

**Table 2:** AuNPs-based systems for pathogen antibiotic susceptibility characterization, preclinical/clinical metadata of sensitivity and specificity.

Pathogen	Target	Sensitivity	Specificity	No. of isolates	Reference
MRSA	Detection of mecA gene associated with methicillin resistance, in S. aureus. Genomic DNA samples isolated from cultured bacterial cells	Analytical sensitivity of 66 pg/μl of MRSA total genomic DNA	n.d.	n.d.	Storhoff et al. (2004)
	Detection of mecA gene associated with methicillin resistance, in S. aureus. Validation with clinical samples	97.14% (Compared with culture standard culture methods)	91.89% (Compared with culture standard culture methods)	72	Chan et al. (2014)
MDRTB	Detection of rpoB mutations associated Rifampicin resistance	84.7% (compared to NNO-LiPA Rif. TB assay)	100% (compared to NNO-LiPA Rif. TB assay)	46	Veigas et al. (2010)
	Detection of *rpo*B mutations associated Rifampicin resistance. Integration with isothermal DNA amplification strategies	100% ( compared to NNO-LiPA Rif. TB assay)	100% ( compared to NNO-LiPA Rif. TB assay)	12	Veigas et al. (2013)
	Detection of *rpo*B and *inhA* mutations associated with Rifampicin and Isoniazid resistance. Integration with surface-anchored rolling circle amplification for isothermal DNA amplification. Validation with clinical samples	Analitycal sensitivity of 8.2 pg uL^-1^ of genomic DNA from clinical samples	n.d.	5	Xiang et al. (2013)
	Detection of rpoB and inhA mutations associated with Rifampicin and Isoniazid resistance. Integration with multiplex amplification strategy. Validation with clinical samples	100% (compared to NNO-LiPA Rif. TB assay)	100% (compared to NNO-LiPA Rif. TB assay)	25	Pedrosa et al. (2014)

In this case, Storhoff et al. (2004) proposed the use of AuNPs for the colorimetric detection of antibiotic resistant *S. aureus* strains back in 2004 via a colorimetric “spot- and-read” assay for the detection of *mecA* in MRSA genomic DNA samples. In this assay, nucleic acid targets are recognized by DNA-modified Au-nanoprobes that undergo a color change that is visually detectable when solutions are spotted onto an illuminated glass waveguide. This scatter-based method enabled the detection of nucleic acids while demonstrating a remarkable sequence specificity that allowed discrimination of single-base mismatches, deletions or insertions (Storhoff et al., 2004). The method relies on the cross-linking approach targeting the bacterial mecA gene with a limit of detection of 33 nM. This approach was effective in discriminating genomic DNA samples of MRSA from methicillin-sensitive *S. aureus* (MSSA) strains, where the detectable color change was observed only for MRSA with very short hybridisation times (Storhoff et al., 2004). The use of scatter light analysis coupled to the molecular identification approach greatly enhanced detection sensitivity (∼4 orders of magnitude) compared to previously reported absorbance-based spot test, thus enabling detection of zeptomole amounts of DNA target. This sensitivity is possible in a homogeneous format because aggregate formation is detectable even when only a very small fraction of the nanoparticle probes is involved in the hybridisation, suggesting a large change to both color and intensity of scattered light from the complexes (Storhoff et al., 2004).

Recently, Chan et al. (2014) reported the use of AuNPs for direct colorimetric PCR detection of MRSA in clinical specimens. The colorimetric assay comprised probes functionalised with specific oligonucleotides targeting *S. aureus 23S rRNA* and *mecA* sequences. In this study, 72 clinical samples were tested, including positive blood culture, urine, respiratory samples, as well as wound swabs, pus and body fluid. Using conventional bacterial culture as gold standard, the sensitivity, specificity, positive and negative predictive values of this colorimetric assay were 97.14, 91.89, 91.89, and 97.14%, respectively. This performance compares to that of commercial real-time PCR assays but at lower cost per reaction. The colorimetric assay also demonstrated very good agreement with the “gold standard” (94.44%). This study was the first report on the use of AuNPs colorimetric assay for direct detection of MRSA in various types of clinical specimens (Chan et al., 2014).

Further evaluation of these assays in large-scale trials is needed which can also allow for some modifications to streamline the procedures for routine use.

#### AuNPs for molecular characterisation of antibiotic resistance in *M. tuberculosis*

Tuberculosis is one of the leading causes of infection in humans, causing high morbility and mortality all over the world. At present, the treatment of choice for an active TB infection is long-term antibiotic therapy, with an initial “intensive phase” consisting of the four first-line anti-TB drugs (isoniazid, INH; rifampicin, RIF; ethambutol, ETH; and pyrazinamide) followed by a typical four month course of RIF and INH alone (Gaspar et al., 2008). Despite effective treatment, due to the length of antibiotic therapy, side effects frequently develop and the associated cost is high (Garner et al., 2007; Aspler et al., 2008; Armstead and Li, 2011). These factors correlate to low patient compliance and contribute to the development of drug-resistant bacteria (Armstead et al., 2011). The rate of new cases of multidrug resistant tuberculosis (MDRTB) continues to increase, and due to the difficulty in the management of such infection, it constitutes a serious health problem (World Health Organization [WHO], 2012). The surge of MDRTB has raised awareness towards extreme resistant TB (XRDTB) or even totally resistant TB. In most cases, drug resistance in Mtb has been related to mutations in several *loci* within the pathogen’s genome. The development of fast, cheap and simple screening methodologies is of paramount relevance for the early detection of these mutations, essential for the timely and effective diagnosis and management of MDRTB patients (Barnard et al., 2008; Veigas et al., 2010; Abebe et al., 2011). Resistance to RIF is commonly associated with point mutations within the *rpoB* gene of Mtb whose detection is considered the best early molecular predictor for MDRTB. Resistance to RIF has been associated to single point alterations within a well-defined 81 bp region (codons 507–533) of the *rpoB* gene encoding for the beta subunit of RNA polymerase. Concurrent resistance to INH and RIF is commonly associated with point mutations in *katG*, *inhA,* and *rpoB* genes of MTBC (Musser, 1995; Soini and Musser, 2001). Prompt diagnosis of MDRTB has been the main obstacle to its correct management and control. This problem would seem to have been solved with the development of molecular techniques applicable also in high-prevalence, low-income settings, such as the Genotype MTBDR- Plus and Gene Xpert MTB/RIF assays. However, though very rapid and highly sensitive, these tests are not considered highly specific for the diagnosis of RIF resistance, particularly in low prevalence settings or when mixed strains are present (Van Deun et al., 2013).

Based on the differential non-cross-linking aggregation of Au-nanoprobes, Baptista’s group developed a simple and straightforward colorimetric method for Mtb identification and single base mutation discrimination in *rpoB* (Veigas et al., 2010), which constitutes the first application of AuNPs for the specific detection of RIF resistant Mtb. This approach uses an Au-nanoprobe assay for the rapid detection of MTBC strains and simultaneous characterisation of mutations associated with RIF resistance, namely mutations in codons 516, 526, and 531 of *rpoB* gene from MTBC clinical specimens with remarkable sensitivity in a few hours. To assure high selectivity and sensitivity, two nanoprobes are simultaneously used to tackle each mutation – one recognizing the wild-type sequence and another for the mutated. By doing so, this approach correctly detected the presence of DNA from members of the MTBC in 83.3% of all tested samples. The initial approach required a simple PCR amplification of a large region spreading the targets sequences for the nanoprobes, and the resulting amplicons tested directly with the Au-nanoprobe system. The molecular characterisation step takes only 15 min to yield a colorimetric result that, through the use of a suitable photodetector (e.g., UV/visible spectrophotometer, microplate reader, etc.) allows for medium throughput analysis at a peripheral laboratory. A limit of detection could be set at 75 nM, however, for robust single base mismatch determination, 117 nM of DNA target were used per assay (see also **Table 2**).

More recently, the same group extended and improved this detection strategy towards the simultaneous discrimination of specific mutations within *inhA* and *rpoB* genes in PCR amplified DNA from isolates. Using a multiplex PCR reaction, it was possible to assess both *loci* in parallel, and extend the potential of the Au-nanoprobe method to MDRTB molecular characterisation with special application in the most frequent Portuguese genotypes (Pedrosa et al., 2014). Based on the molecular signatures of susceptibility of MTBC members to first line antibiotics, RIF, INH, and ethionamide (ETH), a two-step approach was developed, based on the multi *loci* PCR amplification of gene fragments and subsequent hybridisation with specific Au-nanoprobe. The two target sequences harbor the most common mutations associated with resistance to these antibiotics, *rpoB* S531L, *inhA* C(-)15T and are amplified by a set of *rpoB* primer pairs flanking unique regions specific for MTBC members – first level of identification. The MTBC Au-nanoprobe constitutes a second level of identification. Another two sets of nanoprobes are used to discriminate the desired mutations. This approach brings new possibilities for MDRTB diagnostics as the Au-nanoprobe methodology may become an useful tool for MDRTB molecular characterisation at a point-of-need (Pedrosa et al., 2014).

Conventional TB diagnosis methods (such as Ziehl-Neelsen or Kinyoun for staining sputum smears, egg-based media for culture, and solid media for antimicrobial susceptibility testing) have been used for almost 50 years presenting low sensitivity, specificity, and a high turn-around time. Although some laboratories use fluorochrome stains and liquid-based media for cultures, small hospitals or clinics cannot use these methods due to the need of high technical expertise, equipment, and expensive materials. The quality of sputum specimens and contamination of specimens due to inappropriate storage and/or long transport times to the laboratory has been a critical bottleneck (Wilson, 2011).

The development of complete, accurate and simple TB diagnostic tests able to target relevant TB sequences and assessing multidrug resistance cases has been one of the major bottlenecks for TB effective detection and treatment. Au-nanoprobes integrated within a paper-based platform may be proven to be an accurate, rapid, low-cost, and user friendly nanosystem for the identification of specific DNA sequences of TB, confirming infection and allowing identification of MDRTB strains. This nanosystem allows earlier treatment, reduction of time spent on inappropriate and ineffective treatment and reduction of MDRTB spread in congregate settings making it ideal for large screening and/or at point-of-need. The ability of coupling the LAMP amplification strategy to specific Au-nanoprobes translates into additional benefits: it eliminates the PCR amplification step bypassing the need of specialized machines and technicians, assessing multiple antibiotic resistances in the field and reaching remote communities.

***Integration of isothermal amplification techniques to AuNPs strategies.*** Isothermal amplification techniques have been recently developed as an alternative to PCR for target DNA amplification and detection without the use of a thermocycler (Gill and Ghaemi, 2008). Thermophilic helicase-dependent isothermal amplification uses a thermostable helicase to unwind the double stranded DNA (dsDNA) and generate single stranded templates that are used for further polymerase amplification (Lixin et al., 2005). The dsDNA separation and amplification are performed at the same temperature, which makes this technique suitable for development of point-of-care microbial detection systems, since a thermocycler is not required for DNA denaturation and amplification (Tomita et al., 2008; Jeong et al., 2009). Recently, a AuNP based DNA biosensor for the detection of Mtb using thermophilic helicase-dependent isothermal amplification was developed (Torres-Chavolla and Alocilja, 2011). In recent years, Loop-mediated isothermal amplification (LAMP, Eiken Chemical Co. Ltd., Tokyo, Japan) assay has been introduced for the diagnosis of pulmonary TB (Yuan et al., 2013). The general LAMP procedure uses four primers to achieve a cyclical amplification process based on spontaneous formation of stem-loop DNA structures. This process also utilizes a polymerase with strand displacement capability. LAMP has been used to successfully detect a wide range of pathogens including malaria, HIV, and multiplexed detection of bacteria (Hartman et al., 2013).

Further improvements to the Au-nanoprobe system described above were attained via LAMP amplification strategy coupled to specific Au-nanoprobes for molecular identification of MTBC members and resistance signatures, such as RIF resistance (Veigas et al., 2013). Taking advantage of such features, they demonstrated that the non-cross-linking system is capable to discriminate the *rpoB* S531L point mutation on LAMP products and, thus, opening new possibilities for MDRTB diagnostics in remote environments and at a point-of-care. LAMP originates long DNA concatamers that can easily be assessed via a set of nanoprobes for individual sequence identities, demonstrating that it is possible to use an Au-nanoprobe based strategy to detect single point alteration on isothermally amplified DNA products.

Despite the several benefits presented by these nanodiagnostics systems, translation into the clinics is still unaccomplished. Most of the TB nanosystems reported in the literature still lack validation and for most of them integration in one simple platform capable of eliminating the need for DNA purification and amplification is of utmost importance. Refinement of these laboratory strategies into one single nanodevice may speed up translation into the field.

***Rolling circle amplification.*** Following on the development of isothermal amplification methods, Xiang et al. (2013) developed a surface-anchored rolling circle amplification (RCA) integrated with Au-nanoprobes to isothermally detect multiple point mutations associated with MDRTB with a wild-type to mutant ratio of 5000:1. This work introduced a new SPR method for multiplex mutation detection based on surface signal amplification. The high sensitivity and specificity of this method mainly attributed to the high-fidelity of ligation, multiplexing characteristics of probes, amplification potential of surface-anchored RCA and Au NPs, and intrinsically high sensitivity of SPR biosensor. The L-RCA by ligase relies on base pairing principle which requires perfect complementarity on the ligation nick. It not only forbids the mismatch but also has a low occurrence of false positive results when compared to PCR (Lizardi et al., 1998). Because RCA amplifies only the circular PLP without accumulation of target templates over time, it minimizes the risk of contamination and the potential biohazard. Besides, the Au-nanoprobes further enhance identification due to the sandwich hybridisation. Upon recognition, each point mutation is identified by locating into the corresponding channel on a chip, which allows the immobilized primer (capture probe)–template (circular PLP) complex to isothermally amplify as RCA and further amplified by AuNPs. Binding of the AuNPs to the RCA products acts as the electromagnetic field coupling to the gold film, thus enhancing the plasmon resonance derived by excitation by the incident light, leading to improvement of the transduction of small changes in refractive index on the chip surface media, thus improving sensitivity (Petryayeva and Krull, 2011).

## NANODIAGNOSTICS FOR POINT OF CARE APPLICATIONS

Despite the amazing advances of nanotechnology the effective translation to the clinical setting and to the molecular detection and/or characterisation has not been fully applied (Hauck et al., 2010). Nanotechnology, and NPs, based molecular identification systems have focused on increasing sensitivity and speed when compared to traditional methodologies. However, nowadays researchers have been gearing their efforts towards the development of nanotechnology-based systems that are affordable, robust and reproducible, making them suitable for applications even in areas that lack dedicated and expensive laboratory equipment. In fact, AuNPs based systems have been proposed and used for the identification of different pathogens with one common ground – making it simple and affordable. Considering that most of these systems rely on the molecular recognition of selective and specific sequences in DNA, we are only one step away from identifying molecular signatures of resistance. In fact, only by bringing together these platforms and those at the forefront of antibiotic resistance characterisation (e.g., microbiologist and clinicians), definite translation can be achieved. Technology integration together with the possibility of miniaturization is of utmost importance for the development of an integrated biosensor suitable for peripheral laboratories and/or point-of-care diagnostics, providing a new tool in the fight against TB.

Nonetheless, there has been some effort towards bringing these technologies to point-of-care application. For example, the Au-nanoprobe system for characterisation of mutations associated to drug resistance in TB has been further integrated with a paper-based platform for fast and easy to use detection of MTBC members – *Gold on Paper* (Veigas et al., 2012b; Costa et al., 2014). *Gold on Paper* is the working concept of integrating a paper micro well platform and a biomolecular detection scheme based on Au-nanoprobes. *Gold on Paper* showed to be capable of efficiently detect MTBC members directly and, by means of a smartphone device, analyzing data on the spot while maintaining sensitivity and specificity. This demonstrates that systems such as *Gold on Paper* may be easy to perform without the need for expensive and complex laboratory set up. Using this concept, it is possible to attain a positive identification of the pathogen within one hour, which via the use of a generic “smart” mobile device allows for complete analysis at a peripheral laboratory, and transmit digital information over existing communications channels, combined with GPS location metadata inserted into the captured digital images (Veigas et al., 2012b). The limitation imposed by the DNA sample preparation is greatly overcome by the potential use of this methodology to identify and characterize the molecular signatures involved in antibiotic resistance. This integrated diagnostics scheme can then forward the attained data to a centralized off-site server allowing for monitoring of TB in real-time that could be proven extremely useful in remote areas of the globe lacking resources (Veigas et al., 2012b).

Monitoring for drug-induced liver injury (DILI) via serial transaminase measurements in patients on potentially hepatotoxic medications (e.g., for HIV and TB) is routine in resource-rich nations, but often unavailable in resource-limited settings. Towards enabling universal access to affordable point-of-care screening for DILI, Pollock et al. (2013) have performed the first field evaluation of a paper-based, microfluidic finger-stick test for rapid, semi-quantitative, visual measurement of blood alanine aminotransferase. The objective was to assess operational feasibility, inter-operator variability, lot variability, device failure rate, and accuracy, to inform device modification for further field testing. The paper-based alanine aminotransferase test was performed at point-of-care on fingerstick samples from 600 outpatients receiving HIV treatment in Vietnam (Pollock et al., 2013). This first field study performed with a paper-based microfluidic device opens the door to development of similar assays for other important analytes and also for assessing MDRTB and MRSA.

Based on these principles new technologies were developed and are today available in the market. For example, Nanosphere offers two products approved by the FDA, one aimed at identifying typical mutations in coagulation factors without the need for nucleic acid amplification; another used to genotype polymorphisms associated with warfarin metabolism. In both cases the samples are processed through a cartridge where the sample analyzed via an automated processor and reader (Lefferts et al., 2010; Maurice et al., 2010).

## CONCLUSIONS AND FUTURE PERSPECTIVES

Over the past decades, noble metal NPs, due to their optical and physic-chemical properties, have been used in proof-of-concept biosensing tools for the sensitive detection of pathogens of interest. Amongst these biosensing platforms, several have focused on the specific identification of DNA/RNA sequences associated to molecular signatures of infection and antibiotic resistance. AuNP based assays have progressively been integrated into sensing platforms capable of increasing sensitivity and lowering costs. Here, we provided an overview of existing strategies relying on the use of AuNPs for detection of molecular markers of antibiotic resistance. Despite the desperate need for robust, yet simple and cheap, screening tools to identify MDR pathogens, there are not that many concepts making it through to validation in the laboratory set. It is clear that microbiologists need to integrate the multidisciplinary teams that provide for nanodiagnostics development so as to widen the scope of combinations and modalities that can be easily coupled to current molecular nanodiagnostics technologies so as to facilitate integration to the lab and clinical setting.

Detection strategies based on AuNPs provide comparable detection capability to that of standard techniques but at a fraction of cost and time, usually not requiring cumbersome sample preparation or equipment. As such, nanoparticle based approaches are expected to be incrementally applied to MDR characterisation and pathogen detection with particular emphasis for systems capable to operate at point-of-need. However, despite the massive investment in these technologies, translation to the clinics is yet to be fulfilled. Most of the reported systems in the literature still lack validation and/or are in pre-clinic stages with few commercially available products being available to the clinician. The next step is clearly to focus on the translation of some of the strategies that exist in the lab into the field and to the bedside.

## Conflict of Interest Statement

The authors declare that the research was conducted in the absence of any commercial or financial relationships that could be construed as a potential conflict of interest.
